# A novel allogeneic acellular matrix scaffold for porcine cartilage regeneration

**DOI:** 10.1186/s12896-023-00800-x

**Published:** 2023-09-14

**Authors:** Huiming Jiang, Jun Lu, Jiawei Li, Zizheng Liu, Fufei Chen, Rui Wu, Xingquan Xu, Yuan Liu, Yiqiu Jiang, Dongquan Shi

**Affiliations:** 1 Department of Sports Medicine and Joint Surgery, Nanjing First Hospital, Nanjing Medical University, Nanjing, 210000 Jiangsu PR China; 2 State Key Laboratory of Pharmaceutical Biotechnology, Division of Sports Medicine and Adult Reconstructive Surgery, Department of Orthopedic Surgery, Affiliated Drum Tower Hospital, Medical School, Nanjing University, 321 Zhongshan Road, Nanjing, 210008 Jiangsu PR China; 3 State Key Laboratory of Pharmaceutical Biotechnology, Division of Sports Medicine and Adult Reconstructive Surgery, Department of Orthopedic Surgery, Nanjing Drum Tower Hospital, The Affiliated Hospital of Nanjing University Medical School, Nanjing, 210008 Jiangsu PR China

**Keywords:** Cartilage repair, Acellular matrix scaffold, Tissue engineering, Regenerative medicine

## Abstract

**Background:**

Cartilage defects are common sports injuries without significant treatment. Articular cartilage with inferior regenerative potential resulted in the poor formation of hyaline cartilage in defects. Acellular matrix scaffolds provide a microenvironment and biochemical properties similar to those of native tissues and are widely used for tissue regeneration. Therefore, we aimed to design a novel acellular cartilage matrix scaffold (ACS) for cartilage regeneration and hyaline-like cartilage formation.

**Methods:**

Four types of cartilage injury models, including full-thickness cartilage defects (6.5 and 8.5 mm in diameter and 2.5 mm in depth) and osteochondral defects (6.5 and 8.5 mm in diameter and 5 mm in depth), were constructed in the trochlear groove of the right femurs of pigs (n = 32, female, 25–40 kg). The pigs were divided into 8 groups (4 in each group) based on post-surgery treatment differences. was assessed by macroscopic appearance, magnetic resonance imaging (MRI), micro–computed tomography (micro-CT), and histologic and immunohistochemistry tests.

**Results:**

At 6 months, the ACS-implanted group exhibited better defect filling and a greater number of chondrocyte-like cells in the defect area than the blank groups. MRI and micro-CT imaging evaluations revealed that ACS implantation was an effective treatment for cartilage regeneration. The immunohistochemistry results suggested that more hyaline-like cartilage was generated in the defects of the ACS-implanted group.

**Conclusions:**

ACS implantation promoted cartilage repair in full-thickness cartilage defects and osteochondral defects with increased hyaline-like cartilage formation at the 6-month follow-up.

## Introduction

Cartilage defects are a common sports injury observed in 60% of patients who undergo arthroscopic procedures [1]. Unfortunately, troublesome cartilage regeneration has led to unsatisfactory results with current therapeutic strategies for cartilage defects. Articular cartilage with inferior regenerative potential results in poor regeneration of cartilage, especially in hyaline cartilage formation in the defect [2]. The main symptoms of articular cartilage defects are pain and immobility, which further induce the development of osteoarthritis [3]. To achieve cartilage repair in situ, defect filling and formation of hyaline cartilage are required [4]. In recent years, cartilage tissue engineering has been used to attempt cartilage regeneration in situ, but overcoming immunogenicity, simulating the in vivo microenvironment, and performing mechanical repair are difficulties in achieving cartilage repair [5]. The natural cartilage matrix provides a suitable repair microenvironment and a high degree of biocompatibility [6–8]. Among them, acellular cartilage matrix biomimetic scaffolds (ACS) provide tensile strength, connect the framework of cartilage, and affect cell type disposition [9]. Therefore, we focused on designing a novel acellular cartilage matrix scaffold to overcome those flaws for cartilage repair in situ in the present study.

Recently, acellular matrix was verified as an effective scaffold for cartilage regeneration because of the biomimetic microenvironment supporting histogenesis [10]. Thus, the advantages of the acellular cartilage matrix are the highest degree of histocompatibility, native three-dimensional structures, and various bioactive components. Furthermore, compared with alternative scaffolds, ACS induce the recruitment of autologous bone marrow mesenchymal stem cells (BMSCs) in situ [11, 12]. The effect of ACS in different cartilage tissues, such as the auricular, airway and nucleus pulposus, has been reported [13–15]. In this study, a novel ACS is a sponge-like graft produced by hyaline cartilaginous matrix. The ACS possesses a microporous structure similar to native hyaline cartilage [16]. As a special cartilage sponge, a more suitable and more available scaffold is provided by macropores [17]. Thus, ACS easily recruits host chondrocytes or BMSCs across the cartilage-defect interface and repairs the defect, activating the formation of hyaline-like cartilage in situ and promoting interface integration [18].

ACS are divided into three types by the origins of the matrix, which consist of autologous ACS, allogeneic ACS, and xenograft ACS [19]. Several limitations in autologous ACS remain to be solved [20, 21], such as difficulty in cultivation, prolonged production cycle and excessive cost [22]. In addition, xenograft ACS of knee cartilage have been studied in cartilage defects in cartilage engineering, but allogeneic ACS have not yet been studied [23–25]. The characteristics of allogeneic ACS are that they are easier to produce and apply on a large scale [26]. Thus, we believe that the novel allogeneic ACS is a satisfactory and cost-effective strategy for cartilage repair.

In addition, to validate the safety and efficacy of ACS for cartilage and osteochondral repair, a larger animal model is more suitable for simulating human cartilage defects on a variety of sides, including the defect specifications, operation procedure, and usage of scaffold [27]. To verify the clinical indications, full-thickness cartilage defects (2.5 mm in depth) and osteochondral defects (5 mm in depth) are proposed. Furthermore, we set up two depths (6.5 and 8.5 mm diameter) to evaluate whether the different sizes of defects influenced the repair effect of ACS.

In the present study, we designed allogenic ACS to repair defects of varying sizes in a clinically relevant porcine model. We found that ACS promote the formation of hyaline cartilage and subchondral bone remodeling and that different specifications of defects may affect the treatment of ACS implantation.

## Methods

### Preparation of porcine acellular scaffold (ACS)

Allogenic ACS were prepared as previously described [28–31]. The porcine articular cartilage used in this study was provided by TAIHE Biotechnology (Jiangsu, China) (SCXC(SU)2017-0010). Briefly, porcine articular cartilage was cut into 1 mm thick pieces, and then the cartilage sheet was soaked in 1% sodium hydroxide solution for 4 h and crushed and soaked in 3 mol/L hydrochloric acid solution for 48 h [32]. The cartilage digestion solution was digested with 0.2% pepsin solution at 4 °C for 48 h. After that, the precipitate was collected by high-speed centrifugation (2000 rpm, 25 °C, 10 min), and then the precipitate was filled onto the well plate with distilled water to produce the collagen sponge in a vacuum freeze-drier. Then, the different specifications of collagen sponges were immersed in cross-linking solution for 22 h. The cross-linking solution consisted of 1-ethyl-3-(3-dimethylaminopropyl)-carbonized diimide hydrochloride (EDC, 50 mM, Aladdin E106172-100 g) and N-hydroxysuccinimide (NHS, 8 mM, Aladdin H109330-100 g), dissolved by stirring in 95% ethanol solution (37 °C) (Aladdin 64-17-5). Finally, different specifications of ACS were obtained after washing, vacuum freeze-drying, packaging and irradiation sterilization. The ACS contained residual DNA concentrations of 6.28 ng/mg, which were tested by Pico green dsDNA quantitation reagent (Yeasen Biotechnology (Shanghai) Co., 12641ES04) according to Chinese pharmaceutical industry standards (YY/T 0606.25–2014. Tissue engineered medical product-Part 25: Quantification of remnant DNA in biological materials utilizing animal tissues and their derivatives: Fluorescence method.

### Study design and surgical procedure

**Fig. 1:**
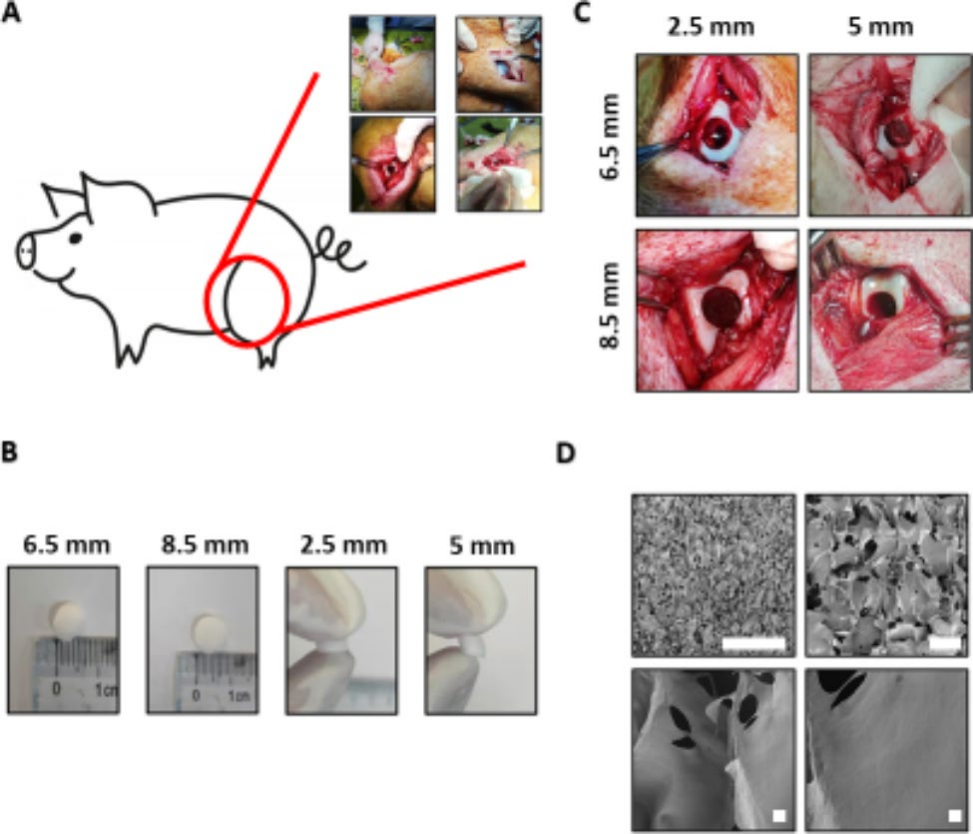
**Schematic illustration of the operation and characterization of the ACS**. (**A**) Schematic illustration of the surgical procedure for cartilage defect repair and ACS implanted. (**B**) Full-thickness cartilage defect model and osteochondral defect model in the medial femoral trochlear: full-thickness cartilage defect, 8.5 mm diameter; full-thickness cartilage defect, 6.5 mm diameter; osteochondral defect, 8.5 mm diameter, 5 mm in depth; osteochondral defect, 6.5 mm diameter, 5 mm in depth. (**C**) Schematic illustration of the ACS. (**D**) Electron microscopic images of the ACS microstructure (Bar: 2 mm, 100 μm, 20 μm, 5 μm)

The protocols used for the collection and analysis of porcine articular cartilage were approved by the institutional laboratory animal ethics committee of Affiliated Drum Tower Hospital, Medical School, Nanjing University (2020AE05003), and the experimental procedures were carried out in accordance with the National Institutes of Health Guide for the Care and Use of Laboratory Animals. The animals used in this study were provided by TAIHE Biotechnology (Jiangsu, China) (SCXC(SU)2017-0010) and were maintained by the laboratory animal center of the Affiliated Drum Tower Hospital, Medical School, Nanjing University. The study included 32 mature female minipigs (Bama minipig, 40 ± 10 kg; 64 knees) that were vested in 1 of 2 conditions: ACS implantation groups or untreated groups (blank groups) (Fig. 1A). Full-thickness chondral defects with intact subchondral bone plates (6.5 or 8.5 mm in diameter) or osteochondral defects with penetrating subchondral bone plates (6.5 or 8.5 mm in diameter; 5-mm depth) were prepared in the trochlea femoris. The eight groups are untreated (blank) groups (6.5 diameter defect, full thickness chondral defect), untreated (blank) groups (8.5 diameter defect, full thickness chondral defect), untreated (blank) groups (6.5 diameter defect, osteochondral defect), untreated (blank) groups (8.5 diameter defect, osteochondral defect), ACS implantation groups (6.5 diameter defect, full thickness chondral defect), ACS implantation groups (8.5 diameter defect, full thickness chondral defect), ACS implantation groups (6.5 diameter defect, osteochondral defect), ACS implantation groups (8.5 diameter defect, osteochondral defect).The detailed surgical approach and defect preparation method were demonstrated in a previous work [33]. All animals were allowed to move freely after the operation. All animals were scarified with potassium chloride solution (10%) after anaesthesia with propofol at 6 months postoperatively. In conclusion, 32 minipigs were randomized into 2 treatment groups: ACS implantation groups and untreated groups (blank treatment).

### Macroscopic assessment

The method of macroscopic assessment was followed by our previous study [34]. All pigs were sacrificed by intravenous propofol overdose under intravenous anesthesia at 6 months postoperatively. The femoral condyles were harvested and photographed without soft tissue. Then, the degree of defect repair was assessed by the ICRS gross scoring system, which included macroscopic appearance, integration to border zone, and overall repair assessment. The scoring was completed by 3 blinded observers.

### MRI Acquisition and assessment

The method of MRI scans was followed by our previous study [34]. At 3 or 6 months after the operation, 3.0-T MRI (Philips) (pulse sequence, FSE; echo time, 44.76 MS; repetition time, 2290 MS) were utilized to acquire knee images before sacrifice. MRI images exhibited the maximum cross section of the defect regions in the sagittal plane. The WORMS scoring system, which included cartilage signal and morphology, was used to analyses the degree of cartilage degeneration and joint injury [35], subarticular bone marrow, and bone. The scoring was performed by 3 blinded observers.

### Micro–computed tomography

The micro-CT scan method was performed according to our previous study [33]. The specimens of the distal femur were fixed in 4% PFA, and then the microstructure of the sections was analyzed using a micro-CT scanner (mCT80; Scanco Medical AG). The scanner was set at a voltage of 70 kV, a current of 114 µA, and a resolution of 15.6 μm per pixel. The quantification analysis of 3D reconstruction images was acquired with Scanco Medical software.

### Histological assessment

The harvested samples were fixed in 4% (v/v) paraformaldehyde for at least 48 h, and then decalcification was completed with formic acid-decalcification solution for 1 month. After dehydration, the specimens were embedded in paraffin and cut into 5-µm coronal sections. Sections of each tissue were assessed by H&E staining and Safranin O/Fast Green staining. The stained sections were observed under an optical microscope (Zeiss). The histological scores were completed by the 3 blinded observers according to the OOCHAS scoring system.

### Immunohistochemistry assessment

Immunohistochemical staining was performed according to the manufacturer’s instructions [36]. We used primary antibodies against collagen I (ab34710; Abcam) and collagen II (ab34712; Abcam) to incubate serial sections overnight at 4 °C. For immunohistochemical staining, HRP (ab6721; Abcam)-conjugated secondary antibodies were added to the slides and incubated at 37 °C for 1 h. Photomicrographs of sections were captured with an optical microscope (Zeiss, Heidelberg, Germany).

### Statistical analysis

The international cartilage repair society macroscopic score (ICRS), OARSI osteoarthritis cartilage histopathology assessment system score (OOCHAS), whole-Organ magnetic resonance imaging score (WORMS) values were analyzed with a nonparametric test (ANOVA based on ranks with post hoc Kruskal‒Wallis test for pairwise comparisons) and are presented as medians with 95% CIs. The results of the micro-CT quantitative analysis were analyzed by one-way ANOVA with the Bonferroni multiple comparison test and are presented as the mean and standard deviation. GraphPad Prism 8.0.1 (GraphPad Software) was used to perform the statistical analyses. A general linear model was used to evaluate the effects of defect size and type (full-thickness chondral vs. osteochondral) on the therapeutic efficacy of ACS implanted treatment. The general linear model was performed using IBM SPSS Statistics 16 (IBM Corporation). P < 0.05 was significantly different for all tests.

## Results

### Characterization of the ACS

There were four types of defects on the condyle of the femur, including full-thickness cartilage defects (2.5 mm in depth) and osteochondral defects (5 mm in depth) with radii of 8.5 mm or 6.5 mm (Fig. 1C). Subsequently, four types of ACS corresponding to the size of the lesion (Fig. 1B) were implanted into the cartilage defect of Panama pigs, which were identified as the scaffold groups. The general character of ACS was white and compact. Moreover, the electron microscopic images of the ACS showed a porous and grid structure that was similar to the natural cartilage microstructure (Fig. 1D).

### Macroscopic view and ICRS macroscopic scoring

**Fig. 2:**
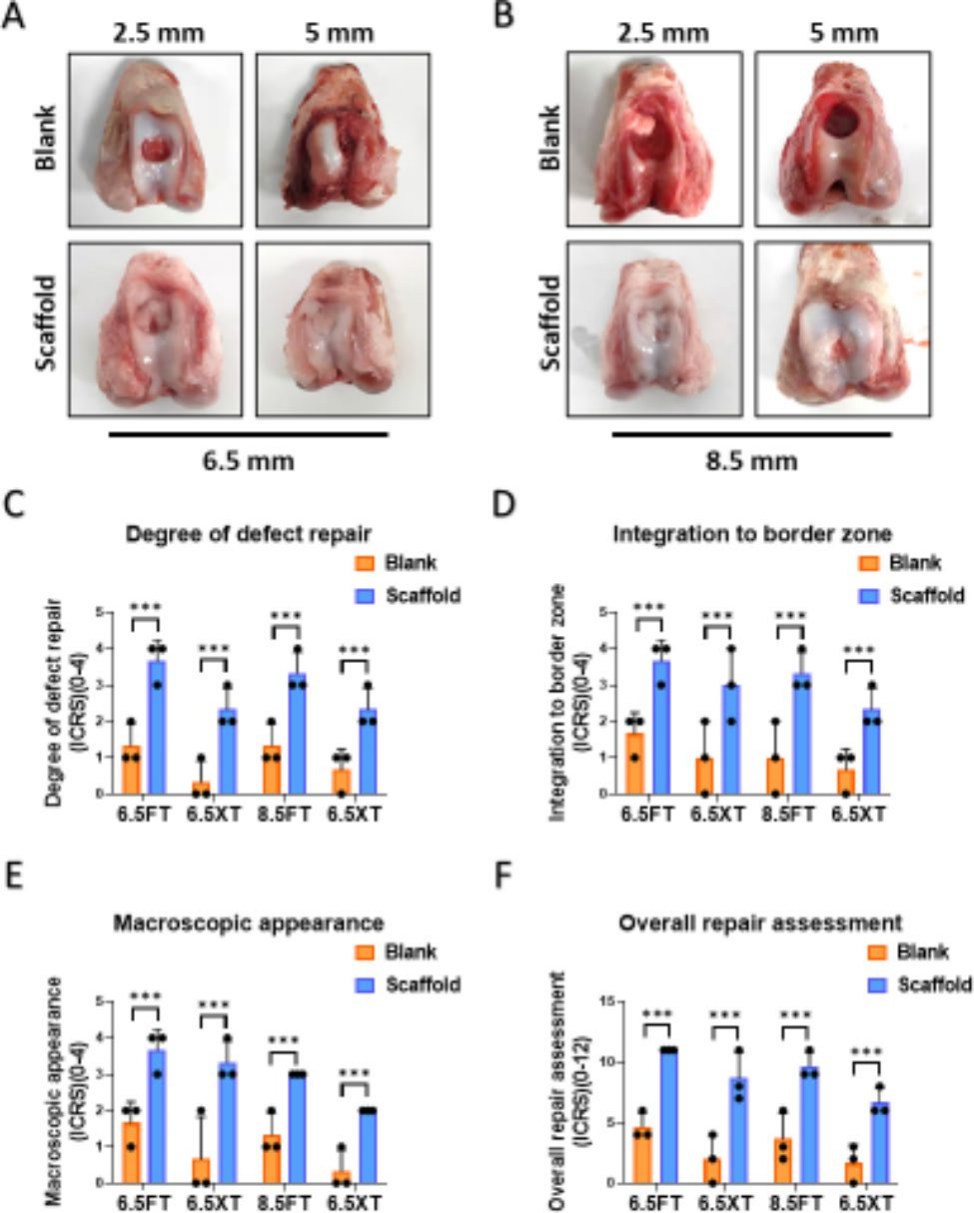
**Macroscopic view and ICRS macroscopic scoring**. (**A**-**B**) The macroscopic appearance of samples at 6 months (**A**) Representative images of macroscopic appearance of the regenerated tissue in 6.5 mm diameter FT or XT models; (**B**) Representative images of macroscopic appearance of the regenerated tissue in 8.5 mm diameter FT or XT models(**C**-**F**) ICRS macroscopic scoring for evaluation of cartilage repair. (**C**) Evaluation of degree of defect repair. (**D**) Evaluation of Integration to border zone. (**E**) Evaluation of macroscopic appearance (**F**) Evaluation of overall repair assessment. (FT: full-thickness cartilage defect; XT: osteochondral defect; Scale bar: 5 mm. The dashed circles indicate original defect boundaries. The ICRS score results are presented as medians with 95% confidence intervals. n = 3, *, P < 0.05; **, P < 0.01; ***, P < 0.001.)

The porcine femoral condyles were harvested 6 months after the operation, and the gross macroscopic view and ICRS macroscopic scoring were used to evaluate the repair effect of ACS. According to the gross macroscopic view, ACS implantation significantly promoted cartilage regeneration at 6 months. The neocartilage showed a normalized macroscopic view with a porcelain white surface and was well integrated with the surrounding native tissue. (Figure. 2 A, B). In contrast, unrepaired defects with fibrillation hyperplasia and cracks were present in the blank groups. ICRS macroscopic scoring analysis was used to evaluate the degree of cartilage regeneration, including the degree of defect repair, integration to the border zone, macroscopic appearance, and overall repair assessment. As shown in Fig. 2C-F, the evaluation showed that the ACS-implanted groups obtained superior scores in each defect type compared to the blank groups.

Furthermore, the depth of the defect influenced the treatment effect of ACS implantation. Obviously, when full-thickness cartilage defects with ACS were implanted, smooth surfaces and slight fissures were formed, which were not observed in osteochondral defects. Moreover, the results in the deeper defect groups showed a low grade of ICRS scoring. However, differences between different diameters under the same depth showed similar regeneration of neocartilage, revealing that the ACS was adapted to defects of different shapes. Together, the macroscopic evaluation revealed that ACS implantation was influenced by the depth of the defect but was independent of the defect radius when the ACS had a suitable size.

### H&E staining of regenerated tissue

**Fig. 3:**
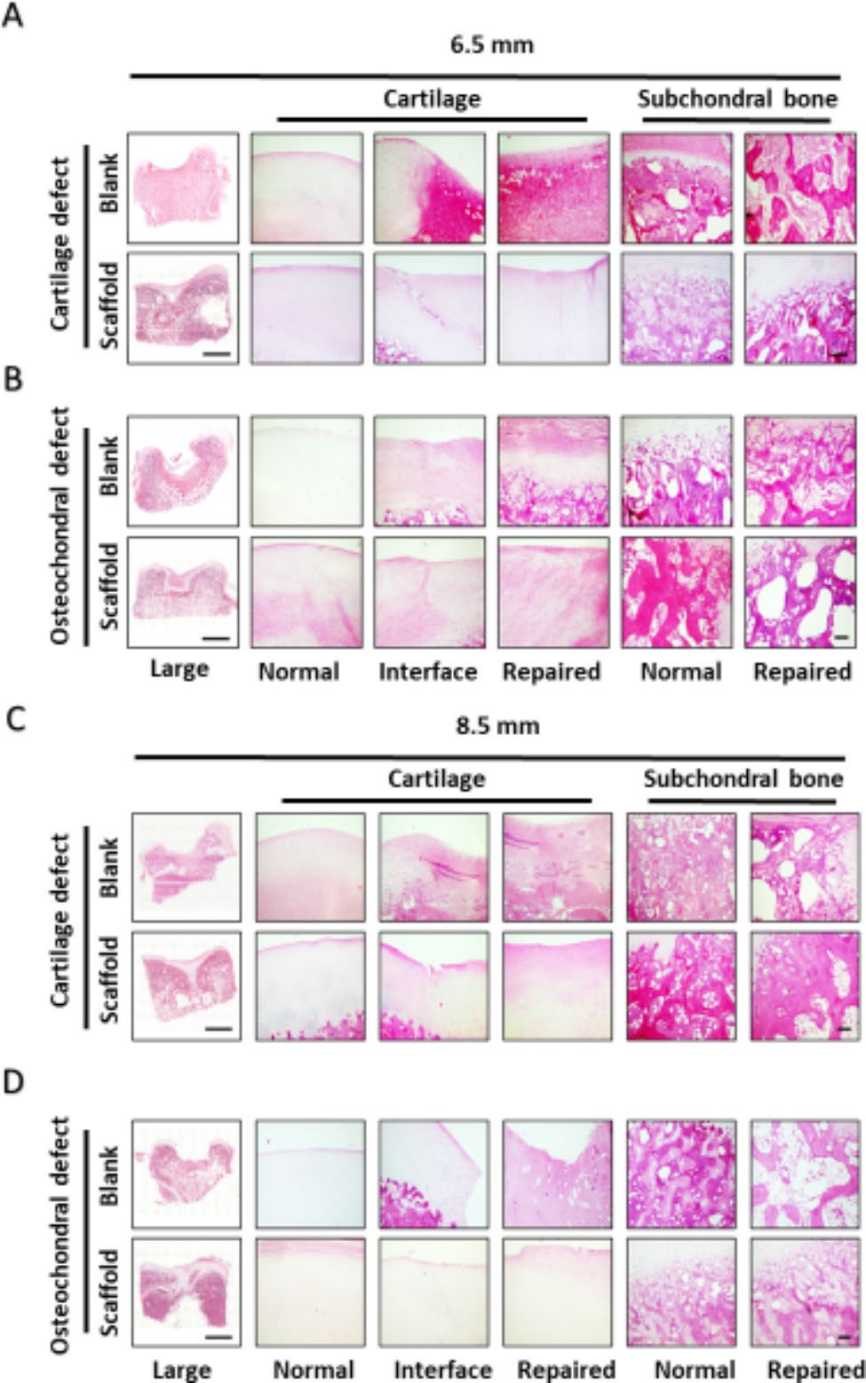
** H&E staining of regenerated tissue showing the cartilage interface, repaired area, and osteochondral bone**. (**A**, **B**) Full-thickness cartilage defects and osteochondral defects of 8.5 mm diameter at 6 months (**C**, **D**) Full-thickness cartilage defects and osteochondral defects of 8.5 mm diameter at 6 months. (Scale bar: 1 mm, 100 μm)

H&E staining was performed to describe the cartilage surface, orientation of collagenous fibers, and subchondral bone reconstruction [37]. The results of H&E staining in the ACS-implanted group revealed superior therapeutic effects in comparison with the blank group in each type of defect. The neocartilage in the ACS-implanted group manifested a smooth cartilage surface, organized collagen fibers, and inconspicuous boundary with the interface area (Fig. 3A-D). In contrast, disorganized fibrocartilage structures with conspicuous boundaries were observed in the blank groups. For subchondral bone, the repaired area resembled normal subchondral bone in the ACS-implanted groups, but trabecular bone disorder was exhibited in the blank group. Similar to the analysis of the macroscopic view, a worse regenerative phenotype was exhibited in the deeper defect, with 8.5 mm diameters revealing a significant difference compared to 6.5 mm diameters.

### Safranin O/Fast green staining of regenerated tissue and OOCHAS scoring

**Fig. 4:**
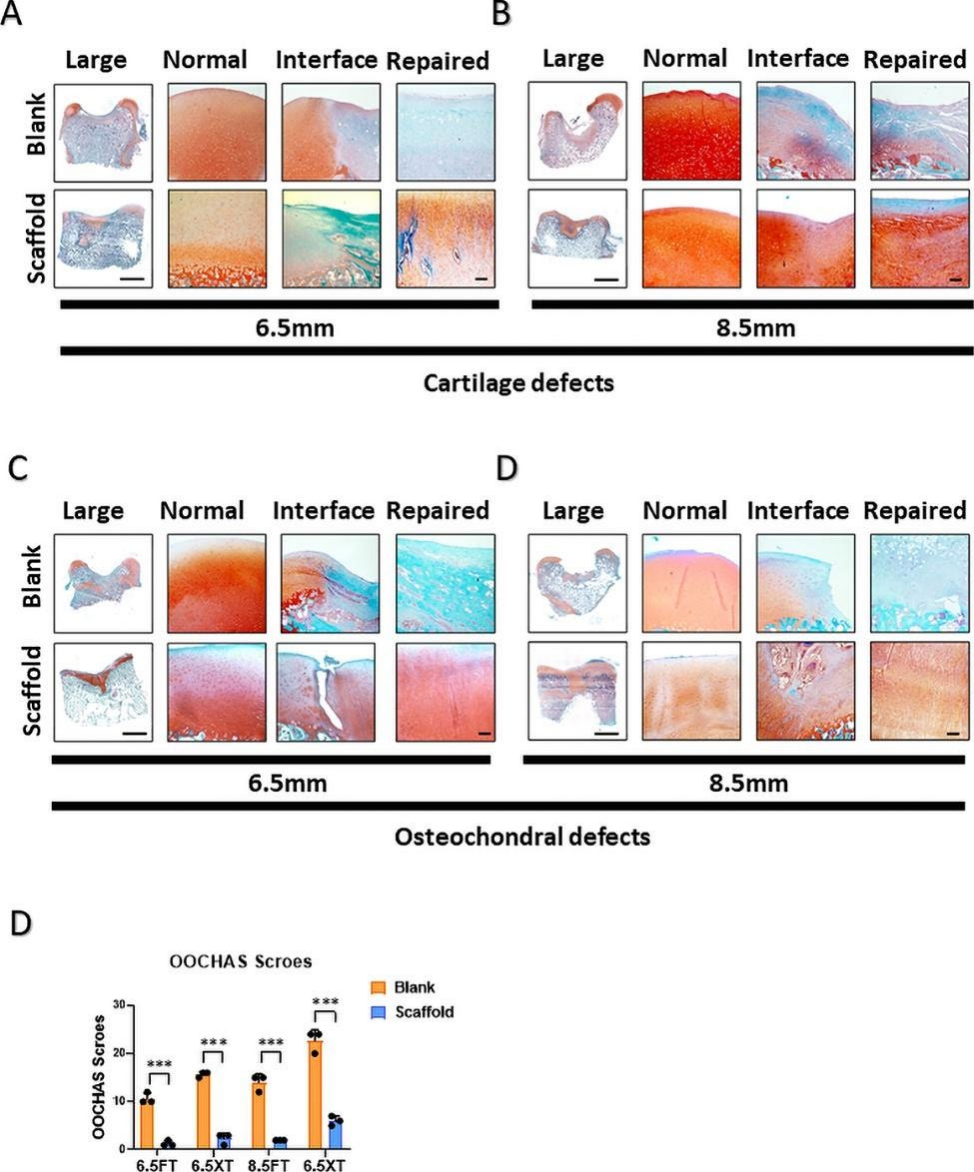
**Safranin O/Fast green staining of regenerated tissue showing the cartilage interface and repaired area**. (**A**, **B**) Full-thickness cartilage defects and osteochondral defects of 6.5 mm diameter at 6 months (**C**, **D**) Full-thickness cartilage defects and osteochondral defects of 8.5 mm diameter at 6 months. (**E**) OOCHAS scoring for evaluation of cartilage repair. (The results are presented as medians with 95% CIs. n = 3, *, P < 0.05; **, P < 0.01; ***, P < 0.001, Scale bar: 1 mm, 100 μm)

Safranin O/Fast Green staining was carried out to evaluate the proportion of GAG content within the extracellular matrix of the blank and ACS groups. While positive GAG content was seen in the ACS group, the blank groups had little safranin O staining in repaired regions (Fig. 4A-D). In comparison with two different diameters, the positive GAG content showed an insignificant difference between 6.5 mm diameters and 8.5 mm diameters. The result of Safranin O/Fast Green staining was evaluated by OOCHAS scoring (OARSI osteoarthritis cartilage histopathology assessment system), which included GAG contents, cellular phenotypes, and condition of extracellular matrix (Fig. 4E). The blank group received a higher score than the ACS-implanted group, which represented inferior reparation without implantation.

### MRI evaluation and WORMS results

**Fig. 5:**
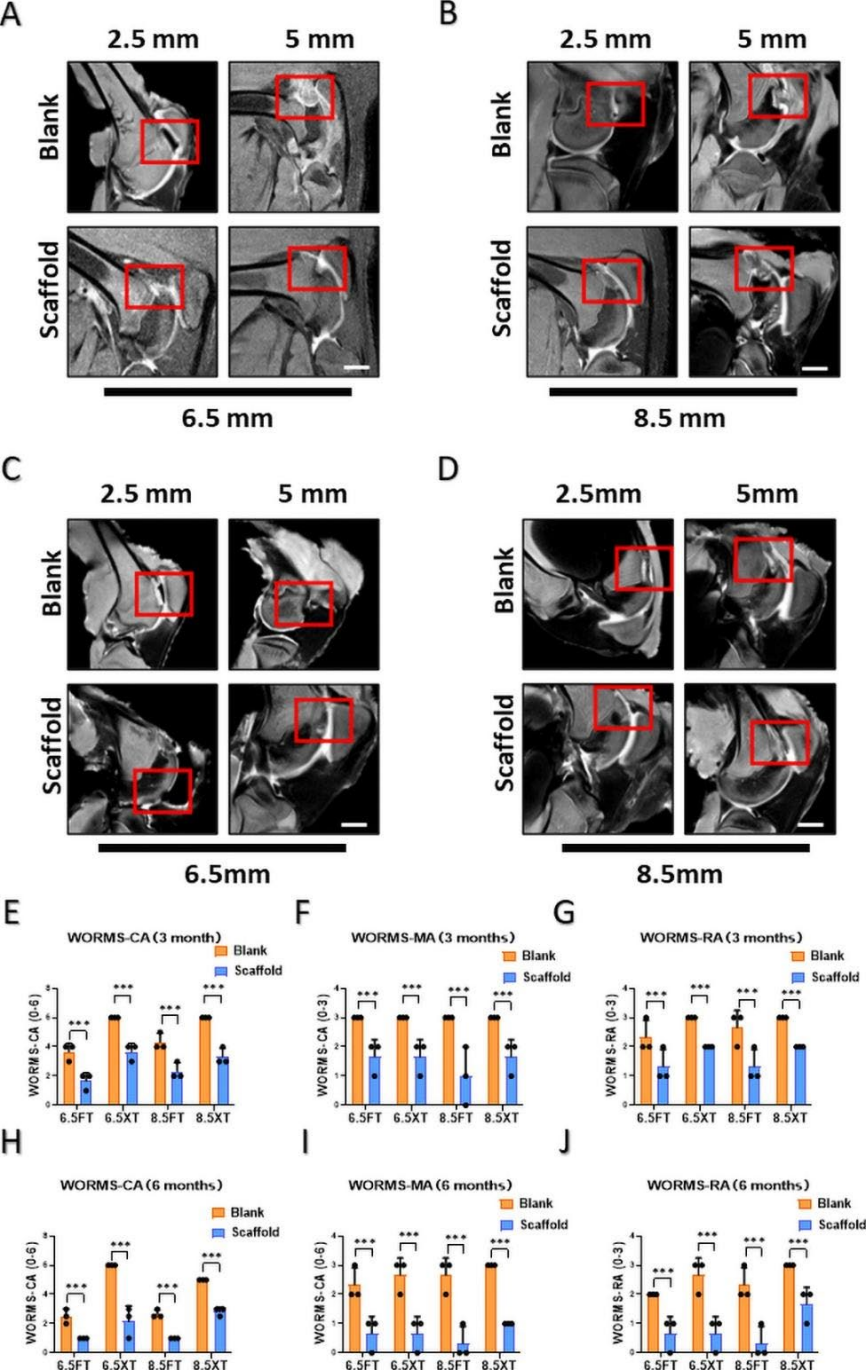
**MRI evaluation and WORMS results**. High-resolution MRI scans of articular cartilage in the sagittal plane at 6 months postoperatively. MRI WORMS results for regenerated tissue at 6 months postoperatively. (**A**, **C**) Full-thickness cartilage defects and osteochondral defects of 6.5 mm diameter at 3 and 6 months respectively. (**B**, **D**) Full-thickness cartilage defects and osteochondral defects of 8.5 mm diameter at 3 and 6 months respectively. (**E**, **H**) WORMS-CA, WORMS Cartilage subscale at 3 and 6 months respectively.; (**F**, **I**) WORMS-MA, WORMS Marrow Abnormality subscale at 3 and 6 months respectively.; (**G**, **J**) WORMS-BA, WORMS Bone Attrition subscale at 3 and 6 months respectively. (The results are presented as medians with 95% Cis. n = 3, *, P < 0.05; **, P < 0.01; ***, P < 0.001, Scale bar: 3 cm)

MRI analysis of full-thickness cartilage defects and osteochondral defect repair was performed at 3 and 6 months. Three months after surgery, the signal intensity of cartilage and subchondral bone in the defect regions approached that of normal tissues nearby in the ACS groups, but visible interfaced lines and bone marrow edema were observed between normal tissue and repaired tissue. (Figure. 5 A-B). Six months after surgery, remarkable improvements in the grade of the defect repair, interface structure, bone marrow edema, and signal intensity of the repair tissue were observed in both cartilage defects and osteochondral defects. By comparison, untreated defects with fibrous hyperplasia were observed in the blank group (Fig. 5 C-D). In contrast to each defect, the interface regions and bone marrow edema were observed in the subchondral defect group with 8.5 mm diameters at 6 months. Furthermore, the quantitative analysis of MRI images by WORMS scores such as cartilage, bone marrow and bone attrition subscale were consistent with images (Fig. 5E-J). The MRI scores for the ACS group were better than those for the blank group in all evaluation indexes. In addition, the WORMS score was higher at 6 months than at 3 months after surgery in all groups.

### micro-CT analysis

**Fig. 6:**
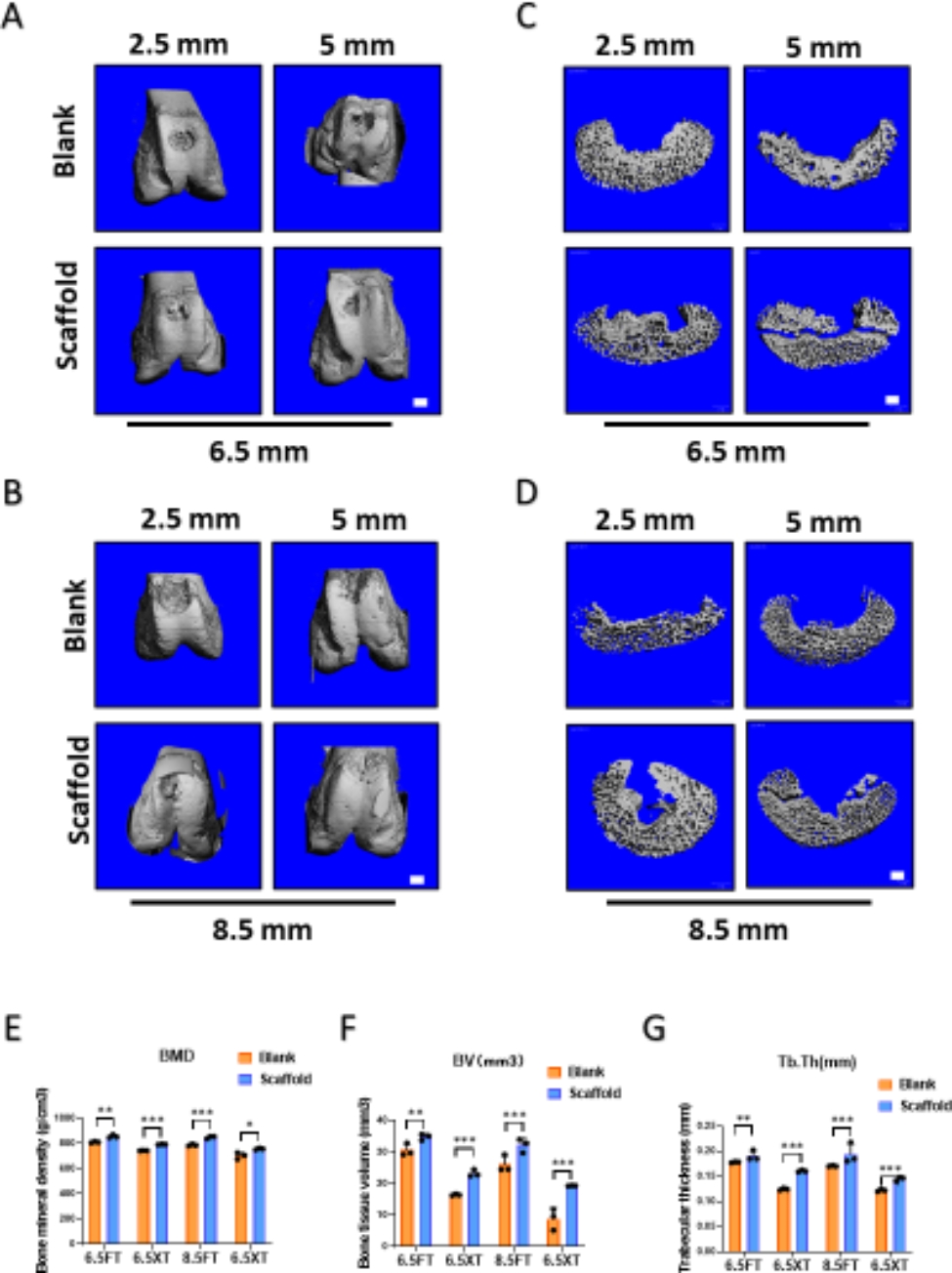
**Micro-Computed tomography analysis**. (**A**-**D**) Assessment of subchondral bone reconstruction in the osteochondral defect group evaluated by micro-computed tomography at 6 months. (**A**, **B**) Three-dimensional reconstruction analysis of full-thickness cartilage defects and osteochondral defects of 6.5 mm diameter and 8.5 mm diameter at 6 months. (**C**, **D**) Cross section analysis of full-thickness cartilage defects and osteochondral defects of 6.5 mm diameter and 8.5 mm diameter at 6 months. (**E**) The quantitative analysis of bone mineral density (g/cm3); (**F**) The quantitative analysis of bone tissue volume (%); (**G**) The quantitative analysis of trabecular thickness (mm). (The results are represented as the mean ± standard deviation. n = 3, *, P < 0.05; **, P < 0.01; ***, P < 0.001. Scale bar: 3 mm.)

The 3D reconstruction and quantitative analysis of micro-CT were utilized to evaluate the degree of subchondral bone reconstruction (Fig. 6A, B). According to 3D reconstruction images of the femoral condyle, all samples manifested varying degrees of subchondral bone regeneration in the ACS-implanted groups. The cross-section of the defect region showed that ACS implantation promoted subchondral bone regeneration (Fig. 6 C, D). Quantitative analysis of micro-CT was further performed, including BMD (bone mineral density), BV (bone volume), and Tb. Th (trabecular thickness) (Fig. 6E-G). Relative to blank groups, ACS–treated defects exhibited a higher percentage of bone volume over the total volume and trabecular thickness in both diameters. However, BMD was not significantly different between the 6.5 mm diameter and 8.5 mm diameter groups but showed a significant difference between the ACS group and blank group.

### Immunohistochemical analysis of Col2 and Col1

**Fig. 7:**
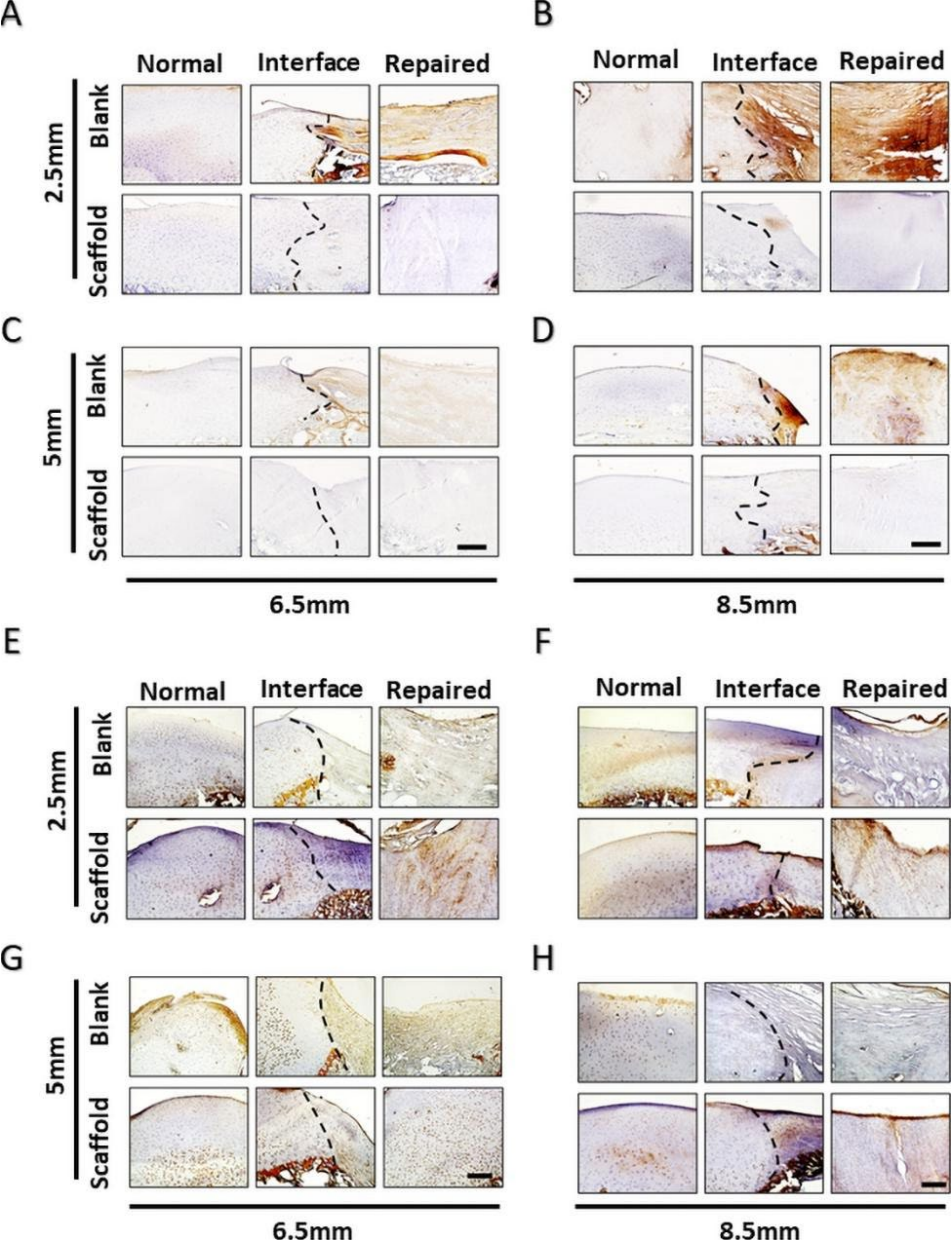
**Immunohistochemical staining of repaired tissue showing Col1 and Col2**. (**A**-**D**) Immunohistochemical staining of Col1 in repaired tissues. (**A**, **B**) Full-thickness chondral defects of 6.5 mm diameter and 8.5 mm diameter in immunohistochemical staining of Col1. (**C**, **D**) Osteochondral defects of 6.5 mm diameter and 8.5 mm diameter in immunohistochemical staining of Col1. (**E**-**H**) Immunohistochemical staining of Col2 in repaired tissues. (**E**, **F**) Full-thickness chondral defects of 6.5 mm diameter and 8.5 mm diameter in immunohistochemical staining of Col2. (**G**, **H**) Osteochondral defects of 6.5 mm diameter and 8.5 mm diameter in immunohistochemical staining of Col2. (The results are represented as the mean ± standard deviation. n = 3, *, *P* < 0.05; **, *P* < 0.01; ***, *P* < 0.001. Scale bar: 200 μm.)

Immunohistochemical analysis of Col2 and Col1 was essential for assessing the cartilage phenotype [37]. Abundant Col2 was present along the repaired tissues and in the ACS groups but not in the blank group (Fig. 7A-D). In contrast, immunohistochemical analysis of Col1 showed low levels in the ACS group but high levels in the blank group in the repaired area (Fig. 7E-H). In addition, the reduction in Col2 was verified in the repaired region in the blank group, showing a dividing line in the interface region in the blank group. For Col1, a similar dividing line indicated that Col1 accumulated in the repaired area without ACS implantation. Thus, the ACS-induced hyaline chondral phenotype was indicated in each model. In summary, ACS is an effective therapeutic approach for hyaline cartilage regeneration.

## Discussion

The results of this study demonstrated that the repair effect of ACS led to cartilage and osteochondral regeneration on the different specifications of cartilage defects. Significant improvements in gross observation and histological grade were revealed in the ACS groups at 6 months post operation. It showed increased hyaline-like cartilage formation and complete interface regions after scaffold implantation. In addition, the ACS group also exhibited normalized neo-cartilage and neo-subchondral bone on MRI and micro-CT imaging.

Hyaline cartilage formation is a standard of successful cartilage regeneration. However, fibrocartilage replaces hyaline cartilage with traditional scaffolds, which results in worse biomechanics and induces osteoarthritis [4]. Neo-cartilage generated by ACS could be considered hyaline-like cartilage, indicating a high proportion of Col2 and a low proportion of Col1 in the present study. The main difference between ACS and conventional scaffolds is that the structure and composition are similar in native cartilage. Apart from the above, previous work has shown that ACS promotes chondrogenic activity, which is suspected to be associated with the recruitment of bone marrow mesenchymal stem cells (BMSCs) [8]. To some extent, ACS makes up for the limited cell supply in the process of cartilage repair [38]. The physiological premise of neo chondrocytes is similar in that native chondrocytes are utilized to produce a matrix to fill a defect, further facilitating a complete interface region. Equivalently, we highlight cell proliferation in the repair areas and interfaced region by ACS implantation. However, that mechanism was not conclusive, and further investigation is needed.

The porcine model plays a vital role in the present study, which is closer to the human clinical situation than other animal models [39]. First, the porcine knee joint exhibits thicker articular cartilage, permitting investigation of both full-thickness cartilage defects and osteochondral defects [40]. Second, pigs maintain a large weight and size, which promote an analogous bearing load and biomechanics in contrast to humans [41]. Third, the porcine model could be evaluated by clinical evaluation criteria such as MRI imaging, showing the repair progress of ACS implantation when animals are alive in the present study [42]. In addition, porcine models have also been utilized to study the effect of allografts on cartilage defects [43]. Together, the porcine model is in favor of exploring clinical indications in our study.

Full-thickness cartilage defects and osteochondral defects represent the most common type of cartilage injury and late stage cartilage defects with osteoarthritis, respectively [12]. Multiple depths of defects require a scaffold that can preserve each and all the structural layers of natural cartilage [5]. In a previous study, simple structural scaffolds showed marked depression in the cartilage surface [11]. To solve this challenge, specific chemical and physical properties of the scaffolds were designed to adapt to the different layered cartilage (47). In the present study, the ACS showed adaptability to different layers in a part of the symbol, especially at a diameter of 6.5 in the osteochondral defect. To evaluate the repair of the osteochondral defect, the complex structure of the interface between cartilage and subchondral bone is more complex [44]. Our results suggest that ACS plays an essential role in repairing the interface between the subchondral bone and cartilage.

Different diameters of defects lead to different therapeutic effects, and several scaffolds have shown restrictions in large defects [45]. In the present study, although the osteochondral defect had lower scores than the full-thickness cartilage defect, the 8.5 mm diameter model showed an insignificant difference compared with the 6.5 mm diameter model. Together, our results indicated that the ACS not only accommodates the different cartilage layers but is also able to repair large defects.

The use of ACS for the repair of cartilage defects has been widely successful. However, several improvements need more study. With the limitations of biocompatibility resolved, xenogeneic scaffolds showed several advantages over allogenic scaffolds, which need to be investigated in the next step [46]. In addition, the mechanism of regeneration of hyaline-like cartilage and subchondral bone were unproven, and it is deemed to advance investigation. Moreover, allogenic ACS was prepared for clinical application in the next investigation.

## Conclusions

According to the present study, ACS implantation treatment promoted the capability of cartilage repair in both full-thickness cartilage defects and osteochondral defects at the 6-month follow-up. ACS treatment might be a good candidate for cartilage injury.

## Data Availability

The datasets used and/or analyzed during the current study available from the corresponding author on reasonable request.

## Abbreviations

ACS: Acellular cartilage matrix scaffold
MRI: Magnetic resonance imaging
BMSCs: Bone marrow mesenchymal stem cells
ICRS: The International Cartilage Repair Society
FT: Full-thickness cartilage defect
XT: Osteochondral defect
OOCHAS scoring: OARSI Osteoarthritis Cartilage Histopathology Assessment System
WORMS: Whole-Organ Magnetic Resonance Imaging Score
GAG: Glycosaminoglycan
BMD: Bone mineral density
BV: Bone volume
Tb. Th: Trabecular thickness
